# Comparative Analysis of Shear Bond Strength of Four Adhesive Systems on Primary Dentin

**DOI:** 10.1002/cre2.70205

**Published:** 2025-08-22

**Authors:** Faezeh Abedi, Sepehr Siahvoshi, Mahdi Babaei, Shima Nourmohammadi

**Affiliations:** ^1^ General Dentist Arak Iran; ^2^ Dental Materials Research Center, Dental School Islamic Azad University of Medical Sciences Tehran Iran; ^3^ Department of Pediatric Dentistry, School of Dentistry Arak University of Medical Sciences Arak Iran

**Keywords:** composite resins, dentin‐bonding agents, materials testing, shear strength

## Abstract

**Objective:**

This study aimed to conduct a comparative evaluation of the shear bond strength of etch and rinse (Single Bond 2), two‐step self‐etch (Clearfil SE) and one‐step self‐etch (Go Bond SDI and G‐Premio Bond) bonding agents applied to primary teeth, using a universal testing machine.

**Material and Methods:**

The sample was divided into four groups: Group A incorporated Single Bond 2, Group B constituted Clearfil SE, Group C constituted Go Bond SDI, and Group D constituted G‐Premio Bond. In each group, 14 out of the total 56 primary canine teeth were considered. Composite restoration was performed using the identified bonding agents on the exposed dentin. A universal testing device was used to evaluate the shear bond strength of the samples. An ANOVA test was used to statistically analyze the obtained data.

**Results:**

By using Kruskal–Wallis test, and determining 0.0080 as the level of significance, shear bond strength with mean standard deviation (SD) was 44.76 ± 32.49 MPa, 92.87 ± 48.63 MPa, 18.55 ± 14.08 MPa, and 22.65 ± 7.74 for Single Bond 2, Clearfil SE, Go Bond SDI, and G‐Premio Bond, respectively. Maximum shear bond strength was found in the two‐step self‐etch bonding agent.

**Conclusion:**

Since dentin is involved in most of pediatric caries, especially in rampant caries, providing an appropriate bond has a great impact on a successful restoration treatment. Considering our results, two‐step self‐etch bonding agent (Clearfil SE bond) demonstrated superior shear bond strength in dentin of anterior primary teeth, over Single Bond 2, Go Bond SDI, and G‐Premio bonds. To establish a correlation among various factors affecting bond efficacy, further in vitro and clinical investigations are necessary.

## 1 Introduction

Carious lesions in primary teeth are extremely common in the pediatric group (Maklennan et al. 2024). Several methods, such as resin‐modified glass ionomer, resin composites, zirconia, and celluloid crowns, have been deployed to repair primary anterior teeth (Thanaratikul et al. 2016). Due to their pronounced durability, exceptional esthetics, favorable adherence to the tooth structure, and ease of application, resin composites have emerged as one of the most prevalently employed materials for the restoration of primary teeth (Bahrololoomi and Mehravar 2022). Following cavity pretreatment with an adhesive system, composite restorations are applied (Swanson et al. 2008). Clinical efficacy of any resin hinges primarily on the degree of interface adhesion and chemical stability that it maintains (Donly and García‐Godoy 2015).

Etch‐and‐rinse and self‐etch systems are available bonding agents that are accustomed to these tasks. First approach involves the use of the acid etching technique, which requires thorough removal of the smear layer and demineralization of intact dentin beneath the surface (Nakabayashi et al. 1982; Pashley and Carvalho 1997). The development of self‐etching systems has bypassed the need for smear layer removal. Since these systems are independent of dentin moisture and do not require rinsing, they facilitate decalcification and resin penetration among the collagen fibers of enamel and dentin, a process that is less complex and less prone to degradation (Ogliari et al. 2006).

The self‐etching system utilizes a solution incorporating the conditioner, primer, and resin, which is simultaneously applied to the enamel and dentin. Marginal seals generated from these materials are akin to, and befitting to those of conventional systems (Davari et al. 2007). For this type of bonding agent, the modality of action involves an uncomplicated process leading to dentin surface demineralization, followed by the simultaneous infiltration of monomers into the created porosities (Agostini et al. 2001). Application procedures have been substantially reduced from three in the universal bonding agents to one in one‐step self‐etch agents (Afshar et al. 2015). Simplicity of the application processes of one‐step self‐etch adhesives, coupled with their swift application and reduced technical sensitivity, render them beneficial for children, especially those with poor cooperation levels. Newer incarnations of these adhesive systems, termed universal adhesives, are versatile and can be employed in total‐etch, selective‐etch, and self‐etch modes (Thanaratikul et al. 2016; Uekusa et al. 2006).

Primary teeth exhibit a wider diameter and a denser configuration of dentinal tubules. Moreover, they are less mineralized and provide a lesser bonding surface than permanent teeth. These considerations spark concerns regarding the bond strength and durability of primary teeth restorations, and impact the ease with which dentin smear layers can be removed (Bahrololoomi and Mehravar 2022; Krämer et al. 2014; Krämer and Frankenberger 2004; Van Meerbeek et al. 2011).

Shear bond strength (SBS) test is the most frequently employed laboratory measure for assessing efficiency of dentin bonding agents, indicating that it serves as a substantial source of dental adhesion data (Burke et al. 2008). Low SBS correlates with inadequate bonding and larger gaps between resin restorations and the tooth.

Chopade et al. (2016) and Gateva and Dikov (2012) demonstrated that two‐step self‐etch bonding systems exhibit superior shear bond strength (SBS) compared to one‐step self‐etch systems. Additionally, Yaseen and Subba Reddy (2009) and Senawongse et al. (2004) independently reported no statistically significant differences in bond strength between self‐etch (one‐ and two‐step) and total‐etch bonding agents. Given the conflicting findings from prior research in pediatric populations (Chopade et al. 2016; Gateva 2012; Yaseen and Subba Reddy 2009; Senawongse et al. 2004; Nakabayashi et al. 1982; Tay and Pashley 2002), this study aims to evaluate and compare the SBS of etch‐and‐rinse and various self‐etch adhesives to dentin in this setting.

The null hypothesis was considered as nonsignificant difference between the SBS of self‐etch adhesives and etch and rinse agent. This evaluation was conducted on anterior primary teeth that had been extracted, and the testing was performed using a universal testing machine (Uekusa et al. 2006).

## 2 Materials and Methods

This study was conducted after obtaining written consent and receiving ethical approval (IR.ARAKMU.REC.1397.029) from Arak University of Medical Sciences. 56 primary canine teeth were visually inspected and identified as healthy, non‐carious, and devoid of any fractures, structural defects, or prior restoration attempts, and were subsequently extracted to proceed with orthodontic treatment. They were randomly divided into four groups, based on a block randomization, each comprising 14 teeth (Table 1). All specimens were mounted by the root section within a self‐curing acrylic substance (Acropars200 cold cure acrylic, Iran) that measured a diameter of 2 cm and a length of 4 cm, such that the entire dental crown extended outside the acrylic. The dimensions of these acrylic cylinders were determined to ensure appropriate placement within the bond strength measurement device.

**Table 1:** Classification of patients based on the type of bonding agent applied.

Group A	Group B	Group C	Group D
Single Bond 2	Clearfil SE	Go Bond SDI	G‐Premio Bond

### 2.1 Preparation of the Dental Surface

The longitudinal axis‐aligned drilling by using a diamond fissure bur (Tizkavan, Iran), the enamel was detached from the labial surface of the teeth with a high‐speed handpiece under water‐air cooling. The bur's diameter‐sized thickness penetrated the tooth from its outer surface, ensuring the complete elimination of the enamel layer, by penetrating 1.5 times of bur's diameter (1.5 mm). Each bur was used strictly for the grinding of not more than five teeth. Subsequently, the exposed dentin surfaces were polished using 600‐grit silicon carbide sandpaper (3M, USA). Under a light microscope (MFC‐2, Russia) with a 20‐fold magnification, the samples were meticulously scrutinized to determine the dentin exposure and confirm the absence of any residual enamel.

### 2.2 Execution of the Restorative Procedure

Upon verifying the absolute exposure of the dentin, Group A, employed an etch and rinse agent (3M ESPE Adper Single Bond 2, USA), whereas Group B used two‐step self‐etch bonding agent (Clearfil SE Bond Primer Kuraray Noritake Dental Inc., Japan), the Group C one‐step self‐etch bonding agent (Go Bond SDI, Australia), formerly known as seventh‐generation bonding and Group D another one‐step self‐etch bonding agent (G‐Premio Bond GC corporation, Japan), which was formerly known as eighth generation.

The procedural protocol and duration of application for each material incorporated in this study adhered strictly to the manufacturer's provided catalog and a single operator performed all the bonding procedures. A translucent plastic tubing with 3 × 3 mm² was subsequently filled with microhybrid resin composite (Point 4, Kerr Italia SPA enamel, Italy) and appropriately coated. The suitably prepared dentin was positioned and cured for a duration of 40 s employing a light cure device (Guilin Woodpecker Medical Instrument Co., China) exhibiting an output intensity of 1200 mw/cm². In subsequent movements, surplus resin composites exterior to the defined range of the tube were excised using a surgical blade (Navak, Iran). The plastic tubing was then cautiously detached from the composite utilizing a surgical blade. Consequently, a composite cylinder characterized by a height and diameter of 3 mm is affixed to the labial surface of the tooth. This composite cylinder was then cured from various orientations for a cumulative timeframe of 40 s. Every step delineated within each of the four groupings was conducted at ambient temperature in compliance with the manufacturer's directive. Finally, to prevent dehydration, the specimens were preserved in distilled water at room temperature for 24 h.

### 2.3 Bond Strength Examination

**Figure 1:**
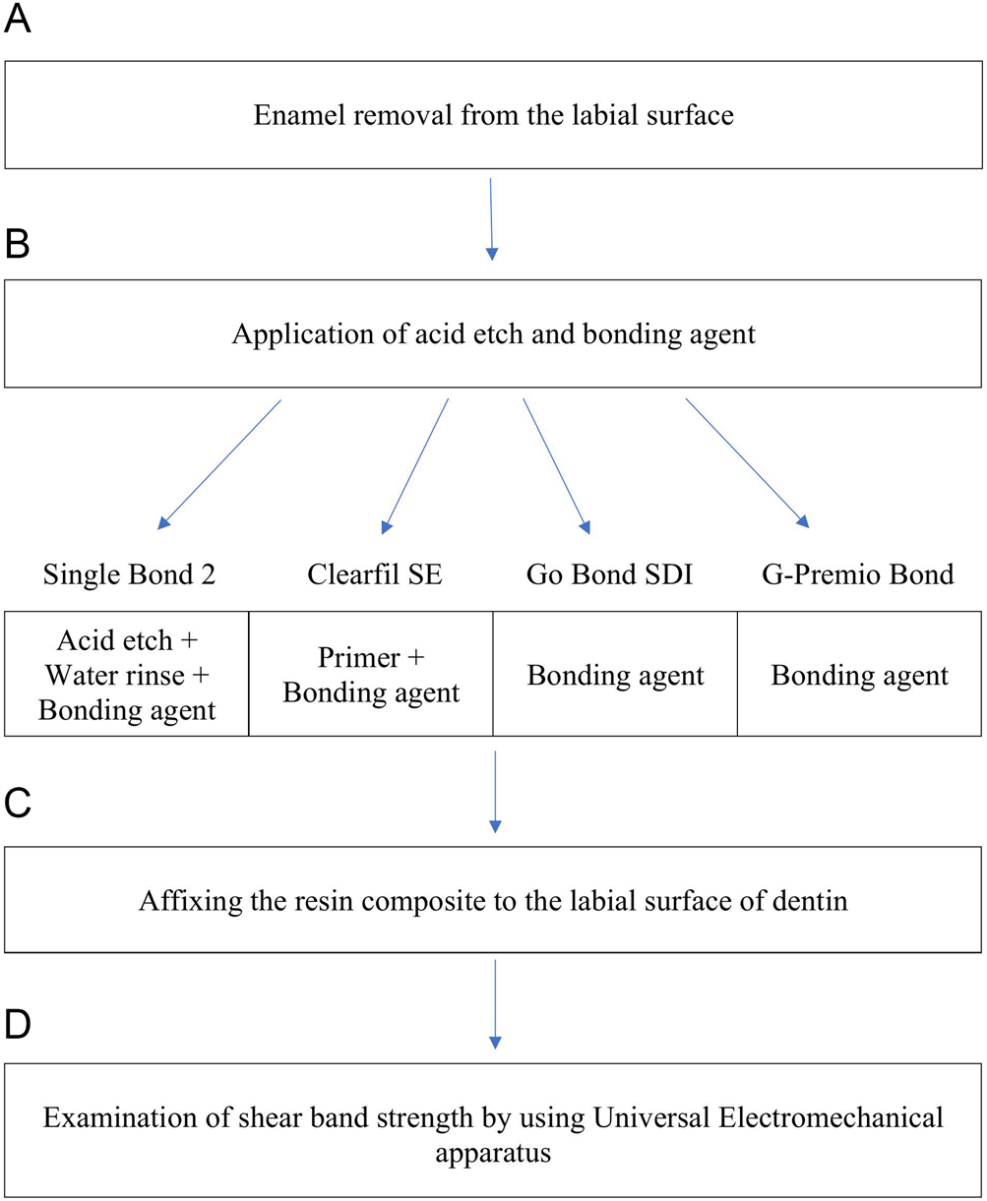
(A‐D) Schematic illustration of the sample preparation protocol.

The samples were evaluated for shear bond strength using Universal Electromechanical apparatus (K–Walter+bai, Switzerland) at a velocity of 1 mm/min, subsequent to the completion of 10000 thermocycling cycles (Dorsa thermocycling, Iran), equivalent to approximately 1 year of clinical use, designed to simulate oral environmental conditions. Concomitantly, all samples were placed in a hot water bath maintained at a temperature of 55 ± 2°C and a cold water bath maintained at a temperature of 5 ± 2°C. Each temperature was held for a duration of 30 s. The force at the fracture point of the composite cylinder was quantified. The average bond strength, represented in MPa, was calculated by dividing the force exerted on the resin composite cylinder by its adhesive cross‐sectional area. Figure 1 summarizes the sample preparation protocol.

### 2.4 Statistical Analysis

The data analysis was conducted using SPSS 16 software. Descriptive statistics, including the mean and standard deviation, were calculated, and a graph was generated to present the data visually. The normality assumption was assessed using the Kolmogorov–Smirnov test. To compare the average shear bond strength between the groups, the Kruskal–Wallis test was utilized. To investigate pairwise differences between groups while minimizing the risk of type 1 error, the Bonferroni correction was employed, with a *P* value of 0.0080 established as the significance level for both the primary analysis and the associated normality and homogeneity of variance tests.

## 3 Results

According to the findings of the study, the average SBS for Group A, 3M‐Adper Single Bond 2, was determined to be 44.76 ± 32.49. For Group B, Clearfil SE, average SBS was calculated to be 92.87 ± 48.63 MPa. In addition, Group C, Go Bond SDI, showed an average value of 18.55 ± 14.08 MPa and average SBS of Group D, G‐Premio Bond was 22.65 ± 7.74 MPa.

The observed differences in mean value of SBS among the four groups were statistically significant (*p* < 0.0001) as depicted in Table 2. Based on these results, it can be concluded that the two‐step self‐etch adhesive, Clearfil SE bond (Group B) from Kuraray Noritake Dental Inc, Japan, exhibited the highest SBS, while the one‐step self‐etch bonding agent, Go Bond SDI (Group C) from Australia, demonstrated the lowest SBS.

**Table 2:** Comparison of mean and standard deviation of shear bond strength in four groups.

Single Bond 2 (*N* = 14)	Clearfil SE (*N* = 14)	Go Bond SDI (*N* = 14)	G‐Premio Bond (*N* = 14)	Kruskal–Wallis test
Mean ± SD	Mean ± SD	Mean ± SD	Mean ± SD	Statistic = 27.07 *p*‐value = 0.0001
44.76 ± 32.49	92.87 ± 48.63	18.55 ± 14.08	22.65 ± 7.74	

The pairwise comparisons yielded statistically significant differences in mean SBS of the evaluated dental bonding agents in most of the comparisons. Specifically, the mean SBS of 3M‐Adper Single Bond 2 (48.10 ± 11.46 MPa) was significantly lower than that of Clearfil SE (*p* = 0.0001). Conversely, Clearfil SE exhibited a significantly higher mean SBS (74.32 ± 11.46 MPa) compared to Go Bond SDI (*p* = 0.0001). Clearfil SE exhibited a significantly higher mean SBS (70.22 ± 11.45 MPa) compared to G‐Premio Bond (*p* = 0.0001); however, the value for 3M‐Adper Single Bond 2 (26.21 ± 11.46 MPa) was not significantly higher than that of Go Bond SDI (*p* = 0.0260) (Table 3).

**Table 3:** Descriptive statistics in four groups.

Groups	Minimum	25th percentile	Median	75th percentile	Maximum
Single Bond 2	14.45	17.41	37.77	53.61	132.02
Clearfil SE	20.90	41.263	99.368	132.05	163.52
Go Bond SDI	5.76	8.40	15.05	20.32	52.49
G‐Premio Bond	12.74	17.39	21.40	23.16	41.66

SEM analysis showed that in Group B fractures occurred within the composite, while Groups A, C, and D exhibited adhesive or interfacial fractures, evidenced by the presence of smear layer and exposed dentin.

**Figure 2:**
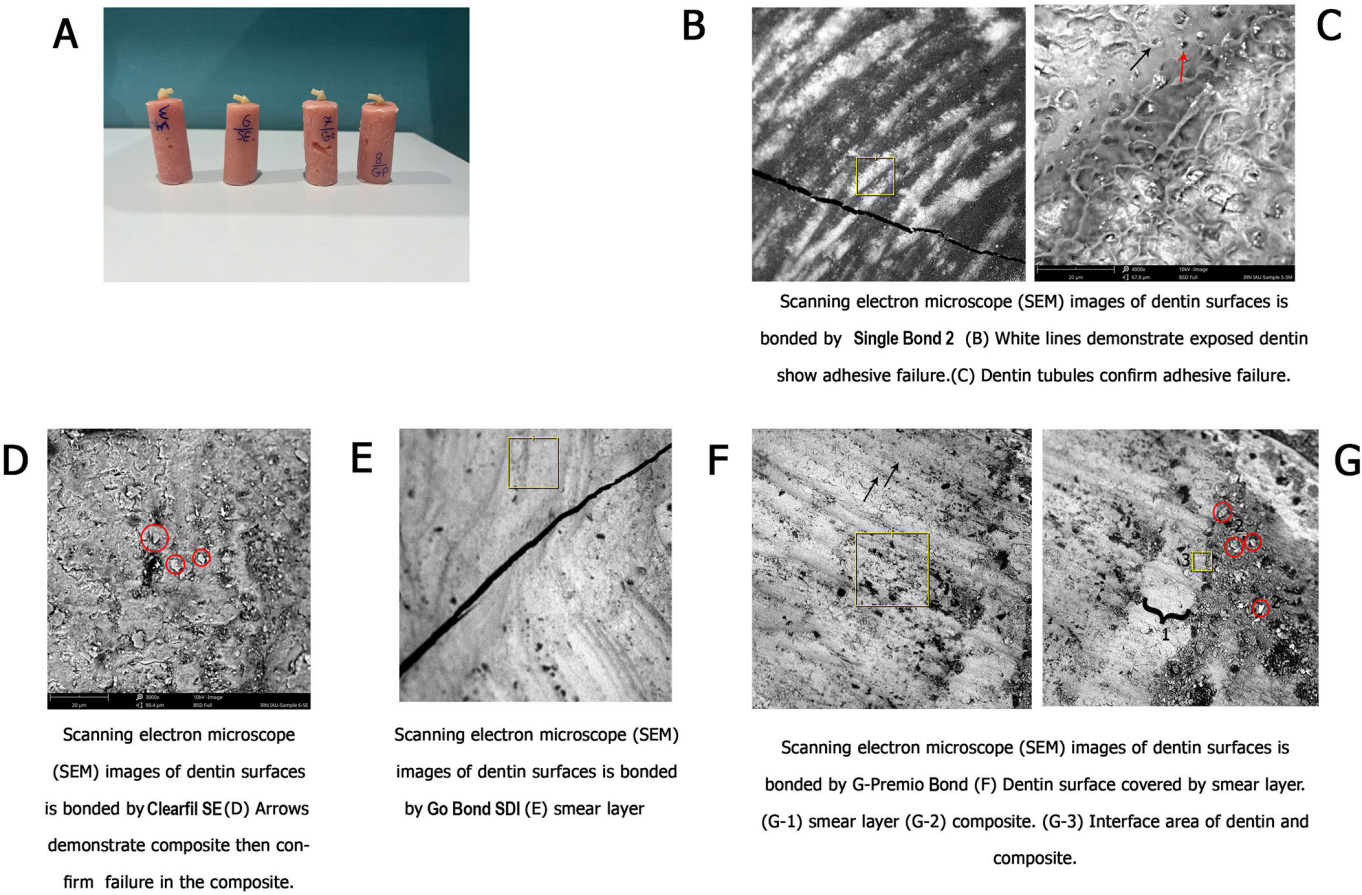
(A) Primary dentin samples. (B‐G) Scanning electron microscope (SEM) images of the dentin surface after bonding by deployed adhesives.

Figure 2 depicts scanning electron microscope (SEM) images of the dentin surface after bonding by deployed agents.

## 4 Discussion

Our results revealed that two‐step self‐etch adhesives provided the highest shear bond strength, followed by etch‐and‐rinse systems, while one‐step self‐etch adhesives showed the lowest values.

There are a few studies (Davari et al. 2007; Chopade et al. 2016; Gateva and Dikov 2012; Yaseen and Subba Reddy 2009; Senawongse et al. 2004) which have examined shear bond strength (SBS) of single‐ versus two‐step self‐etch adhesives on coronal dentin of primary teeth, and producing inconsistent results, however the revelations from the current research harmoniously align with previous studies conducted by Chopade et al. (2016) and Gateva and Dikov (2012), which both reported outperformance of two‐step self‐etch adhesives over one‐step alternatives. This solely can be attributed to a low concentration of the solvent, minimal hydrophilicity, greater extent of polymerization, limited etching, and demineralization of dentin over time (Nakabayashi et al. 1982; Tay and Pashley 2002).

In a pediatric setting, a study by Meharry et al. (2013), a two‐step self‐etch adhesive (Clearfil SE, Optibond FL, Optibond XTR, and Prelude) showed higher SBS than a one‐step self‐etch, which is similar to our results.

Regarding pediatric restorative investigation, some studies evaluate the push out strength of bonding agents to root dentin of primary teeth; i.e., a research by Afshar et al. (2015) that revealed higher but nonsignificant values for two‐step self‐etch adhesive (Clearfil SE) in comparison with etch and rinse (Single Bond 2) and one‐step self‐etch adhesive (Single Bond Universal); however, our study demonstrated that the two‐step adhesive (Clearfil SE) yielded not just higher but significantly higher SBS values.

This discrepancy could be due to a different type of dentin that was examined. Comparing to root dentin, coronal dentin has got a larger amount of inter‐tubular dentin and dentinal tubules are larger, denser, and more divergent and abundant (Afshar et al. 2015). The reason why we examined the coronal dentin is that restoration adhesion, is predominantly provided through the dentin rather than the enamel, especially when most of crown is destroyed by extensive caries.

Nonetheless, another study by Ramos et al. (2016) found that certain self‐etch adhesives, such as CSBP (Clearfil S3 Bond Plus), may achieve high bond strengths to primary dentin, comparable to those obtained with the evaluated etch‐and‐rinse adhesive

Also in a study by Soares et al. (2022), 1 year after placement, the self‐etch adhesives evaluated demonstrated clinical effectiveness comparable to that of the etch‐and‐rinse adhesive for Class II restorations in primary molars.

Our results are in contrast with these two studies which could be attributed to different bonding agent evaluated or preparation protocol; however, this discrepancy further necessitates a need for comprehensive investigation in pediatric setting.

Another study by Soares et al. (2020) demonstrated higher bond strength of the etch‐and‐rinse adhesive (GI‐Prime& Bond XP, PBXP) to primary teeth than that of the two self‐etch adhesives (GII‐Clearfil SE Protect Bond and GIII‐Clearfil S3 Bond Plus), but they evaluated the enamel rather than the dentin.

Despite the Go Bond SDI bonding material's suitability for restorative treatments in children due to fewer process steps, the weakest shear bond strength was observed in this investigation in relation to Group A (etch and rinse) and Group B (two‐step self‐etch) bonding agents. Similar results for Group D (G‐Premio Bond) were obtained, placing it in the second weakest position. Alongside the necessity for substantial bond strength in composite restorations, this arguably advises against its use. We think that placing all components in a single package may interfere with the proper action of each ingredient.

Based on our SEM analysis, the fractures in Group B occurred within the composite material itself, rather than at the interface as observed in the other groups. This provides additional evidence of this adhesive's strength.

In current exploration, the blade of SBS testing apparatus was positioned within a 1 mm interface between the resin composite and the tooth, and force was applied at a rate of 1 mm/min. Applying the force 1 mm above the bonded interface is commonly recommended by ISO and ADA guidelines for consistency across studies. 1 mm height, shear remains the dominant mode of failure versus tensile/bending forces (Soares et al. 2020).

Notably, the self‐etch bonding system exhibits minimized sensitivity post‐application compared with the etch and rinse system, owing to a weaker acid and incomplete elimination of the smear layer. This leads to a decrease in fluid movement toward the exterior of the tubules within the tooth. The self‐etch bonding system, which presents characteristics such as fewer procedural stages, shortened application time, reduced technical sensitivity, and suitability for pediatric dental restorations due to the omission of acid etching and rinsing, ensures superior isolation by the absence of gag reflex in children (Kelechava 2020).

Our findings demonstrate that two‐step self‐etch adhesives yield significantly higher shear bond strength. This enhanced durability is particularly advantageous in pediatric dentistry, where restoration longevity and strength are prioritized over the expediency of one‐step procedures. Consequently, the data challenge the applicability of one‐step self‐etch agents in pediatric restorative dentistry; however, larger‐scale in vivo studies are warranted to corroborate these results.

## 5 Conclusion

Clearfil SE Kuraray Noritake Dental Inc. Japan (two‐step self‐etch agents bonding agent, specifically), demonstrated superior SBS compared to 3M‐Adper Single Bond 2 (etch and rinse), G‐Premio Bond and Go Bond SDI (one‐step self‐etch agents), respectively. The current investigation elucidated that the performance of the etch and rinse bonding agent surpassed that of the one‐step self‐etch bonding agents. However, it is imperative to conduct further in vitro and clinical trials with larger sample sizes and prolonged follow‐up periods to arrive at a conclusive determination.

## Author Contributions

Faezeh Abedi was involved in the conception and design of the work, data collection, drafting of the manuscript, critical revision of the manuscript, and final approval of the version to be published. Sepehr Siahvoshi was involved in data analysis, drafting of the manuscript, critical revision of the manuscript, and final approval of the version to be published. Mahdi Babaei was involved in the conception and design of the work, data collection and analysis, drafting of the manuscript, critical revision of the manuscript, and final approval of the version to be published. Shima Nourmohammadi was involved in data collection, drafting of the manuscript, and final approval of the version to be published.

## Conflicts of Interest

The authors declare no conflicts of interest.

## Data Availability

The data that support the findings of this study are available from the corresponding author upon reasonable request.

## References

[cre270205-bib-0001] Afshar, H. , Y. B. Nakhjavani , S. R. Taban , Z. Baniameri , and A. Nahvi . 2015. “Bond Strength of 5th, 6th and 7th Generation Bonding Agents to Intracanal Dentin of Primary Teeth.” Journal of Dentistry 12, no. 2: 90–98. 26056518 PMC4434132

[cre270205-bib-0002] Agostini, F. G. , C. Kaaden , and J. M. Powers . 2001. “Bond Strength of Self‐Etching Primers to Enamel and Dentin of Primary Teeth.” Pediatric Dentistry 23, no. 6: 481–486. 11800447

[cre270205-bib-0003] Bahrololoomi, Z. , and F. Mehravar . 2022. “Comparison of Different Adhesive Systems on Bond Strength of Resin Composite Posts Placed in Primary Teeth.” International Journal of Dentistry 2022: 1968781. 36072557 10.1155/2022/1968781 PMC9444449

[cre270205-bib-0004] Burke, F. J. , A. Hussain , L. Nolan , and G. J. Fleming . 2008. “Methods Used in Dentine Bonding Tests: An Analysis of 102 Investigations on Bond Strength.” European Journal of Prosthodontics and Restorative Dentistry 16, no. 4: 158–165. 19177726

[cre270205-bib-0005] Chopade, R. V. , P. M. Karade , A. P. Kulkarni , K. S. Bade , A. B. Lavate , and K. V. Chodankar . 2016. “An Evaluation and Comparison of Shear Bond Strength of Two Adhesive Systems to Enamel and Dentin: An In Vitro Study.” Journal of International Oral Health 8: 86–89.

[cre270205-bib-0006] Davari, A. , A. DaneshKazemi , J. Modaresi , Z. Mohammadi , and L. Akbarian . 2007. “The Effect of Light‐Curing Time to Adhesive Layer on Shear Bond Strength of Composite to Dentin.” Journal of Dentistry 8, no. 1: 10–18.

[cre270205-bib-0007] Donly, K. J. , and F. García‐Godoy . 2015. “The Use of Resin‐Based Composite in Children: An Update.” Pediatric Dentistry 37, no. 2: 136–143. 25905655

[cre270205-bib-0008] Gateva, N. , and V. Dikov . 2012. “Bond Strength of Self‐Etch Adhesives With Primary and Permanent Teeth Dentin – In Vitro Study.” Journal of IMAB 2, no. (15): 168–173.

[cre270205-bib-0009] Kelechava, B. 2020. Adhesion Test Methods To Tooth Structure. American National Standard Institute. ANSI/ADA 111‐2019.

[cre270205-bib-0010] Krämer, N. , and R. Frankenberger . 2004. “Restorative Therapy in Deciduous Teeth. Oralprophyl.” Kinderzahnheilkd 26: 78–84.

[cre270205-bib-0011] Krämer, N. , D. Tilch , S. Lücker , and R. Frankenberger . 2014. “Status of Ten Self‐Etch Adhesives for Bonding to Dentin of Primary Teeth.” International Journal of Paediatric Dentistry 24, no. 3: 192–199. 23919411 10.1111/ipd.12059

[cre270205-bib-0012] Maklennan, A. , R. Borg‐Bartolo , R. J. Wierichs , M. Esteves‐Oliveira , and G. Campus . 2024. “A Systematic Review and Meta‐Analysis on Early‐Childhood‐Caries Global Data.” BMC Oral Health 24, no. 1: 835. 10.1186/s12903-024-04605-y. 39049051 PMC11267837

[cre270205-bib-0013] Van Meerbeek, B. , K. Yoshihara , Y. Yoshida , A. Mine , D. M. J., and V. L. K.l. 2011. “State of the Art of Self‐Etch Adhesives.” Dental Materials 27: 17–28. 21109301 10.1016/j.dental.2010.10.023

[cre270205-bib-0014] Meharry, M. , S. Moazzami , and Y. Li. 2013. “Comparison of Enamel and Dentin Shear Bond Strengths of Current Dental Bonding Adhesives From Three Bond Generations.” Operative Dentistry 38, no. 6: E237–E245. 23802638 10.2341/12-521-L

[cre270205-bib-0015] Nakabayashi, N. , K. Kojima , and E. Masuhara . 1982. “The Promotion of Adhesion by the Infiltration of Monomers Into Tooth Substrates.” Journal of Biomedical Materials Research 16, no. 3: 265–273. 7085687 10.1002/jbm.820160307

[cre270205-bib-0016] Ogliari, F. A. , E. Piva , F. F. Demarco , C. S. de Araújo , T. I. da Silva , and S. S. Meireles . 2006. “Microleakage of Seven Adhesive Systems in Enamel and Dentin.” Journal of Contemporary Dental Practice 7, no. 5: 26–33. 17091137

[cre270205-bib-0017] Pashley, D. H. , and R. M. Carvalho . 1997. “Dentine Permeability and Dentine Adhesion.” Journal of Dentistry 25, no. 5: 355–372. 9241954 10.1016/s0300-5712(96)00057-7

[cre270205-bib-0019] Ramos, J. C. , A. D. Soares , S. Torres , A. L. Costa , A. L. Messias , and A. Vinagre. 2016. “Adhesive Interface and Microtensile Bond Strength Evaluation of Four Adhesive Systems to Primary Dentin.” Revista Portuguesa de Estomatologia, Medicina Dentária e Cirurgia Maxilofacial 57, no. 2: 65–73.

[cre270205-bib-0020] Senawongse, P. , C. Harnirattisai , Y. Shimada , and J. Tagami . 2004. “Effective Bond Strength of Current Adhesive Systems on Deciduous and Permanent Dentin.” Operative Dentistry 29, no. 2: 196–202. 15088732

[cre270205-bib-0021] Soares, A. D. , A. L. Costa , L. C. Alves , A. Vinagre , and J. C. Ramos . 2020. “Microtensile Bond Strength of Three Different Adhesive Systems to Primary Enamel: An In Vitro Study.” Pediatric Dentistry 42, no. 6: 476–481. 33369560

[cre270205-bib-0022] Soares, A. D. , J. C. Ramos , L. C. Alves , J. L. Pereira , F. Caramelo , and A. L. Costa . 2022. “Evaluation of the Efficacy of a 2‐Step Etch‐and‐Rinse, 2‐Step Self‐Etch and 1‐Step Self‐Etch Adhesive Systems, in Class II Primary Molars Restorations. An One Year Prospective, Randomized Clinical Trial.” European Archives of Paediatric Dentistry 23, no. 5: 845–854. 10.1007/s40368-022-00748-0. 36098905

[cre270205-bib-0023] Swanson, T. K. , R. J. Feigal , D. Tantbirojn , and J. S. Hodges . 2008. “Effect of Adhesive Systems and Bevel on Enamel Margin Integrity in Primary and Permanent Teeth.” Pediatric Dentistry 30, no. 2: 134–140. 18481578

[cre270205-bib-0024] Tay, F. R. , and D. H. Pashley . 2002. “Dental Adhesives of the Future.” Journal of Adhesive Dentistry 4, no. 2: 91–103. 12236646

[cre270205-bib-0025] Thanaratikul, B. , B. Santiwong , and C. Harnirattisai . 2016. “Self‐Etch or Etch‐and‐Rinse Mode Did Not Affect the Microshear Bond Strength of a Universal Adhesive to Primary Dentin.” Dental Materials Journal 35, no. 2: 174–179. 27041005 10.4012/dmj.2015-109

[cre270205-bib-0026] Uekusa, S. , K. Yamaguchi , M. Miyazaki , K. Tsubota , H. Kurokawa , and Y. Hosoya . 2006. “Bonding Efficacy of Single‐Step Self‐Etch Systems to Sound Primary and Permanent Tooth Dentin.” Operative Dentistry 31, no. 5: 569–576. 17024945 10.2341/05-102

[cre270205-bib-0027] Yaseen, S. , and V. Subba Reddy . 2009, january/March. “Comparative Evaluation of Shear Bond Strength of Two Self‐Etching Adhesives (Sixth and Seventh Generation) on Dentin of Primary and Permanent Teeth: An In Vitro Study.” Journal of Indian Society of Pedodontics and Preventive Dentistry 27, no. 1: 33–38. 19414972 10.4103/0970-4388.50814

